# Psychometric evaluation of the Brief Cognitive Screening Battery: Indonesian version

**DOI:** 10.1590/1980-5764-DN-2024-0250

**Published:** 2025-06-02

**Authors:** Fasihah Irfani Fitri, Dina Nazriani, Octaviasari Agatha Dachi, Ricardo Nitrini, Paulo Caramelli

**Affiliations:** 1 Universitas Sumatera Utara, School of Medicine, Department of Neurology, Medan, Indonesia.; 2 Universitas Sumatera Utara, School of Psychology, Medan, Indonesia.; 3 Universidade de São Paulo, Faculdade de Medicina, Departamento de Neurologia, São Paulo SP, Brazil.; 4 Universidade Federal de Minas Gerais, Faculdade de Medicina, Unidade de Neurologia Cognitiva e do Comportamento, Belo Horizonte MG, Brazil.

**Keywords:** Cognition, Dementia, Indonesia, Psychometrics, Cognição, Demência, Indonésia, Psicometria

## Abstract

Dementia is a significant public health challenge, particularly in low- and middle-income countries such as Indonesia, where underdiagnosis is common.

**Objective:**

To investigate the psychometric properties of the Brief Cognitive Screening Battery – Indonesian Version (BCSB-INA), a culturally-adapted tool aimed at improving the early detection of dementia.

**Methods:**

The tool was tested on a sample of 140 older adults, covering various cognitive domains through multiple subscales. Data were analyzed using bootstrap resampling, Kaiser-Meyer-Olkin (KMO) test, Confirmatory Factor Analysis (CFA), and reliability assessments such as Cronbach's Alpha.

**Results:**

The overall reliability, measured by Cronbach's Alpha, was high (α=0.968). The KMO test yielded a value of 0.889, indicating sample adequacy for factor analysis. The CFA indicated that most dimensions achieved satisfactory validity, with the majority of items demonstrating acceptable factor loadings.

**Conclusions:**

BCSB-INA has demonstrated good reliability and validity throughout its dimensions, suggesting its applicability for use in cognitive assessments in the Indonesian context.

## RESUMO.

A demência constitui um grande desafio para a saúde pública, particularmente em países de renda baixa e média como a Indonésia, onde o subdiagnóstico é comum.

**Objetivo:**

Investigar as propriedades psicométricas da Bateria Breve de Rastreio Cognitivo – Versão Indonésia (*Brief Cognitive Screening Battery* – *Indonesian Version*, BCSB-INA), uma ferramenta culturalmente adaptada que visa melhorar a detecção precoce da demência.

**Métodos:**

O instrumento foi testado em uma amostra de 140 idosos, abrangendo vários domínios cognitivos mediante múltiplas subescalas. Os dados foram analisados por meio de reamostragem de *bootstrap*, teste Kaiser-Meyer-Olkin (KMO), análise fatorial confirmatória (AFC) e avaliações de confiabilidade como o alpha de Cronbach.

**Resultados:**

A confiabilidade geral, medida pelo alfa de Cronbach, foi alta (α=0,968). O teste KMO resultou no valor de 0,889, indicando adequação da amostra para a análise fatorial. De acordo com a AFC, a maioria das dimensões teve validade satisfatória, com cargas fatoriais aceitáveis na maioria dos itens.

**Conclusões:**

A BCSB-INA demonstrou boa confiabilidade e validade ao longo de suas dimensões, sugerindo sua aplicabilidade para uso em avaliações cognitivas no contexto indonésio.

## INTRODUCTION

Dementia poses a significant public health challenge worldwide, particularly in low- and middle-income countries, where it remains significantly underdiagnosed^1^. Timely diagnosis is essential for effective symptom management and for developing appropriate care and support strategies^2^. In response, the World Health Organization (WHO) has established a global action plan with the ambitious goal of ensuring that at least 50% of countries diagnose 50% of their estimated dementia cases by 2025. This initiative emphasizes the pressing need for healthcare systems worldwide to enhance their diagnostic capabilities and develop effective treatment options for individuals affected by this complex condition^3^. Dementia diagnosis still faces barriers, such as stigma, poor access to health care, and limited resources, while facilitators include increased awareness, training for healthcare providers, and family support^4^. A crucial first step in dementia diagnosis is cognitive assessment. This process is not only vital for identifying dementia, but also for monitoring disease progression and evaluating treatment efficacy^5^. The development of culturally- and linguistically-appropriate assessment tools is, therefore, imperative.

In Indonesia, a nation characterized by rich ethnic diversity and varying socio-economic backgrounds, it is essential to have tools that take into account different language proficiencies and levels of education. Many existing standardized tools are predominantly verbal-based, derived from English, and often assume a certain level of education that may not be present in all demographic groups. This creates a barrier to effective diagnosis and care for a significant portion of the population^6^. Efforts to translate or adapt widely-used cognitive tests have been investigated, but they continue to present challenges^7^. For instance, the English idiom "no ifs, ands, or buts" in the Mini-Mental State Examination (MMSE) loses its linguistic complexity, fluency assessment, and cultural significance when translated. Furthermore, ethnic differences in neuropsychological test performance have been acknowledged for some time, regardless of socioeconomic status or educational background^8^.

To address these challenges, it is crucial to adapt existing assessment tools or to create new ones specifically tailored for the Indonesian context. The development of dementia screening instruments must account for cultural nuances, educational disparities, and the need for reliable and valid assessments^6^. While research specifically targeting Indonesia is limited, valuable insights can be gleaned from studies conducted in similar sociocultural contexts^9^. Early detection programs for dementia often employ neuropsychological methods, including two-stage screening processes that assess cognitive deficits^10^. These methods have been efficient in diagnosing dementia in various primary care settings and could be adapted to enhance early identification efforts in Indonesia, given the country's cultural diversity and the challenges posed by low health literacy.

The Brief Cognitive Screening Battery (BCSB) developed by Nitrini et al.^11^ is a well-established tool in Brazil, a country similar to Indonesia in terms of cultural and demographic diversity. Its success in Brazil highlights its potential adaptability to other culturally-diverse settings such as Indonesia. Recent research in North Sumatra has led to the development of the Indonesian version of the BCSB (called BCSB-INA), which has undergone feasibility studies and established normative data among the older adult population in the region^12^. According to the findings, the BCSB-INA is both culturally appropriate and practical for implementation, rendering it a promising tool for dementia screening in both urban and rural areas of Indonesia^12^. Despite these advancements, further research is essential to comprehensively evaluate the effectiveness of the BCSB-INA in distinguishing between cognitively healthy individuals and those with cognitive impairment. This involves investigating its psychometric properties, which are crucial for refining diagnostic practices. Therefore, in this study we aimed to conduct a psychometric evaluation of the BCSB-INA.

## METHODS

### Study design and participants

This cross-sectional study was conducted using non-random consecutive sampling among an older adult population at the Memory Clinic of the Universitas Sumatera Utara Hospital in Medan, Indonesia, from January to August 2024. Eligible participants were adults aged 50 years or older who could effectively communicate in Bahasa Indonesian language. In addition, each participant was required to have an informant — someone who could provide supplementary information regarding the participant's cognitive and functional status — and could provide informed consent to participate in the study. Participants included both individuals with and without a clinical diagnosis of dementia. Those with cognitive complaints or suspected cognitive impairment were evaluated at the Memory Clinic, and their diagnostic status was determined by clinicians based on clinical criteria, cognitive assessments, and informants’ reports. This ensured that the sample included a range of cognitive statuses, allowing for a robust evaluation of the BCSB-INA's psychometric properties in diverse populations. The study was conducted in accordance with ethical guidelines and was approved by the Ethics Committee of the School of Medicine of Universitas Sumatera Utara (No. 540/KEPK/USU/2024). Informed consent was obtained from all participants prior to their involvement in the study.

### Instruments

All participants underwent physical and neurological examinations, along with cognitive assessments using the Indonesian version of the Montreal Cognitive Assessment (MoCA-INA) and the BCSB-INA. The MoCA evaluates various cognitive domains, including visuospatial/executive function, naming, memory, attention, language, abstraction, delayed recall, and orientation to time and place. Visuospatial abilities are assessed through a clock-drawing task and a trail-making task, both of which are useful for evaluating driving fitness. Attention, concentration, and working memory are measured using tasks that involve sustained attention (target detection through tapping), serial subtraction, and forward and backward digit span. Scores range from 0 to 30, with higher scores indicating better cognitive performance. The MoCA awards an additional point for individuals with 12 or fewer years of education^13^. Several adjustments were made to the MoCA-INA compared to the original version, particularly in the assessment of naming, memory, delayed recall, and language functions, due to transcultural validation^14^. The cutoff adjusted to age and level of education in Indonesia was used. Age- and education-adjusted cutoffs specifically designed for the Indonesian context were employed^15^. By accounting for these factors, biases that standard cutoffs might display are reduced, enhancing the relevance and reliability of the cognitive evaluations for identifying impairments in various demographic groups.

### Brief Cognitive Screening Battery

The Brief Cognitive Screening Battery involves presenting a sheet of paper featuring ten simple black-and-white drawings. Participants name each figure (Naming score) and are then asked to recall the drawings immediately, without prior instruction to memorize them (Incidental Memory score). Afterward, the figures are shown again, and participants must memorize them for 30 seconds before recalling them (Immediate Memory score). This process is repeated to assess the Learning score (or encoding). Subsequently, participants complete a Verbal Fluency test (naming animals in one minute) and the Clock Drawing Test (CDT) as interference tasks. They are then asked to recall the previously shown figures (Delayed Recall score). Lastly, a new sheet is presented containing the ten original figures mixed with ten distracting ones, and participants must identify the figures they saw earlier (Recognition score). Scores for these subtests range from 0 to 10 points, except for the Verbal Fluency test^11,16^.

The BCSB was culturally validated for use in Bahasa Indonesian following the ISPOR Principles of Good Practice for cross-cultural adaptation of patient-reported outcome measures. The process involved translating the original English version into Bahasa Indonesian by bilingual translators, followed by reconciliation of the translations, back translation into English, and a review to ensure consistency and accuracy. Discrepancies were addressed through harmonization discussions among translators and researchers. Cognitive debriefing with older adult respondents provided feedback for further refinement, and expert review ensured cultural and linguistic appropriateness. After validation, a feasibility study in North Sumatra was conducted to assess the BCSB-INA's performance and establish baseline cognitive metrics for the older adult population, ensuring its reliability for cognitive status assessment. Detailed findings regarding the cultural validation and feasibility study can be found elsewhere^12^.

### Psychometric testing process

The psychometric testing process of the BCSB-INA involved several critical steps to ensure its reliability and validity. Initially, data were collected through structured interviews and assessments conducted by trained healthcare professionals. This process included a series of cognitive evaluations and questionnaires designed to capture relevant demographic, health, and functional information from participants, providing a comprehensive overview of their cognitive status. The resulting scores from these assessments were then analyzed to evaluate the psychometric properties of the instrument. To prepare the data for analysis, checks for normality were performed, and statistical methods were employed to address any challenges.

The appropriateness of the data for factor analysis was evaluated using the Kaiser-Meyer-Olkin (KMO) measure and Bartlett's test. Internal consistency of the BCSB-INA was assessed using Cronbach's Alpha, and Confirmatory Factor Analysis (CFA) was employed to evaluate its construct validity.

## RESULTS

### Participants’ characteristics

A total of 140 participants completed the cognitive assessment using the BCSB-INA instrument. We present the characteristics of the sample in Table 1. The average age (and standard deviation – SD) of the participants was 59.4±11.1 years. Most participants were women (52.4%), with a large proportion being unemployed, housewife, or retired (73.6%). According to the participants’ educational background, 48.6% had attended college or university.

**Table 1:** Participants’ characteristics (n=140).

Characteristic		Mean (SD)	n	(%)
Age (years)		67.21 (7.61)		
Sex	Men		74	(52.9)
	Women		66	(47.1)
Level of education	Did not attend school		1	(0.7)
	Elementary school		17	(12.1)
	Junior high school		19	(13.6)
	Senior high school		35	(25.0)
	University		68	(48.6)
Occupational status	Unemployed/housewife/retired		99	(70.7)
	Employed		12	(8.6)
	Self-employed		29	(20.7)

As the dataset did not achieve normality (with p<0.05 for most items), bootstrap resampling with one thousand samples was employed to produce more robust estimates. This technique helped to mitigate the impact of non-normality, and the resulting Bollen-Stine bootstrap p-value of 0.336 indicated that the data adequately fit the model after resampling.

### BCSB-INA domain scores

In Table 2 we show the mean scores for the various domains of the BCSB-INA. The mean values and SD for each domain were as follows: Naming 9.24 (2.278); Incidental Memory 4.21 (2.294); Immediate Memory 6.19 (2.840); Learning 6.90 (3.038); Delayed Recall 5.91 (3.511); and Recognition 8.94 (2.685). The overall mean score was 41.38 (14.742), with a Cronbach's alpha of 0.968 for the 60 items.

**Table 2:** Descriptive statistics and reliability.

Domains and Items		Corrected item–total correlation	Mean	SD	Cronbach's alpha	Cronbach's alpha if the item was deleted
Naming	Nm1	0.886	9.24	2.278	0.959	0.953
	Nm2	0.825				0.955
	Nm3	0.825				0.955
	Nm4	0.902				0.953
	Nm5	0.825				0.955
	Nm6	0.840				0.954
	Nm7	0.702				0.962
	Nm8	0.885				0.953
	Nm9	0.855				0.954
	Nm10	0.744				0.959
Incidental memory	Inc_M1	0.273	4.21	2.294	0.651	0.634
	Inc_M2	0.420				0.604
	Inc_M3	0.400				0.607
	Inc_M4	0.226				0.643
	Inc_M5	0.082				0.674
	Inc_M6	0.263				0.637
	Inc_M7	0.300				0.630
	Inc_M8	0.299				0.630
	Inc_M9	0.394				0.609
	Inc_M10	0.502				0.586
Immediate memory	Imd_M1	0.453	6.19	2.840	0.804	0.789
	Imd_M2	0.680				0.767
	Imd_M3	0.667				0.766
	Imd_M4	0.304				0.807
	Imd_M5	0.451				0.790
	Imd_M6	0.448				0.790
	Imd_M7	0.543				0.779
	Imd_M8	0.379				0.798
	Imd_M9	0.447				0.790
	Imd_M10	0.480				0.786
Learning	Ln1	0.626	6.90	3.038	0.861	0.844
	Ln2	0.641				0.842
	Ln3	0.619				0.844
	Ln4	0.523				0.852
	Ln5	0.612				0.844
	Ln6	0.558				0.849
	Ln7	0.456				0.857
	Ln8	0.497				0.854
	Ln9	0.571				0.847
	Ln10	0.636				0.842
Delayed recall	Dly_R1	0.662	5.91	3.511	0.898	0.887
	Dly_R2	0.648				0.888
	Dly_R3	0.677				0.886
	Dly_R4	0.656				0.888
	Dly_R5	0.655				0.888
	Dly_R6	0.582				0.893
	Dly_R7	0.666				0.887
	Dly_R8	0.642				0.889
	Dly_R9	0.581				0.893
	Dly_R10	0.698				0.885
Recognition	Rn1	0.787	8.94	2.685	0.964	0.962
	Rn2	0.857				0.959
	Rn3	0.875				0.958
	Rn4	0.829				0.960
	Rn5	0.881				0.958
	Rn6	0.842				0.960
	Rn7	0.870				0.958
	Rn8	0.881				0.958
	Rn9	0.801				0.961
	Rn10	0.739				0.964
Total score (60 items)			41.38	14.742	0.968	

### Reliability

The reliability of the BCSB-INA was assessed by analyzing the internal consistency of overall score and each domain using all 60 items (Table 2). The overall Cronbach's Alpha result was 0.968, which means that the data have good reliability at a very reliable level of reliability (0.80<α≤1.00). As for the item discrimination power, the item selection criteria on this scale are based on item-rest correlation using the limit *rix*≥0.30. According to the results, there were 58 out of 60 items that met the item discrimination criteria, and there were two items, namely Inc_M4 and Inc_M5, which had slightly lower item discrimination power than the limit used. Items that have good discrimination power are able to distinguish one individual from another according to the attributes measured, so that all items in this study have good quality. In addition, we also carried out a reliability analysis per dimension to see the internal consistency of each dimension.

### Convergent validity

Convergent validity assesses the degree to which a measuring instrument correlates with other tools that measure the same or similar concepts. In this study, we evaluated the convergent validity of the BCSB-INA by examining its correlation with the MoCA-INA. According to the results from the Pearson correlation test, presented in Table 3, the dimensions of BCSB-INA and MoCA-INA showed significant positive correlations, with r values ranging from 0.212 to 0.818, all with p<0.05

**Table 3:** Convergent validity between Brief Cognitive Screening Battery – Indonesian Version and Montreal Cognitive Assessment Versi Indonesia.

	Naming	Incidental memory	Immediate memory	Learning	Delayed recall	Recognition
Visuospatial/executive	r=0.478	r=0.498	r=0.550	r=0.561	r=0.495	r=0.395
	p=0.000	p=0.000	p=0.000	p=0.000	p=0.000	p=0.000
Naming	r=0.657	r=0.518	r=0.565	r=0.587	r=0.550	r=0.523
	p=0.000	p=0.000	p=0.000	p=0.000	p=0.000	p=0.000
Attention	r=0.570	r=0.537	r=0.625	r=0.609	r=0.525	r=0.483
	p=0.000	p=0.000	p=0.000	p=0.000	p=0.000	p=0.000
Language	r=0.447	r=0.438	r=0.521	r=0.536	r=0.490	r=0.418
	p=0.000	p=0.000	p=0.000	p=0.000	p=0.000	p=0.000
Abstraction	r=0.405	r=0.498	r=0.496	r=0.505	r=0.479	r=0.277
	p=0.000	p=0.000	p=0.000	p=0.000	p=0.000	p=0.001
Delayed recall	r=0.212	r=0.379	r=0.390	r=0.381	r=0.407	r=0.245
	p=0.012	p=0.000	p=0.000	p=0.000	p=0.000	p=0.004
Orientation	r=0.617	r=0.757	r=0.810	r=0.818	r=0.811	r=0.623
	p=0.000	p=0.000	p=0.000	p=0.000	p=0.000	p=0.000

Abbreviation: r, correlation coefficient; p, p-value.

### Construct validity

Before proceeding to Confirmatory Factor Analysis (CFA), the Kaiser-Meyer-Olkin (KMO) measure and Bartlett's test were conducted to assess the appropriateness of the data for factor analysis. The KMO value of 0.889 suggested that the sample size was adequate, while Bartlett's test was significant (p<0.05), confirming that the variables in the correlation matrix were not independent (p<0.05) and could be continued with factor analysis. Finally, CFA was performed to evaluate the construct validity of the BCSB-INA. Mixed outcomes were indicated as per the results; while some fit indices, such as the Root Mean Square Error of Approximation (RMSEA=0.08) and Relative χ² (CMIN/DF=1.987), suggested an adequate fit for the model, others, including the Goodness of Fit Index (GFI=0.584), fell below the acceptable threshold.

**Figure 1:**
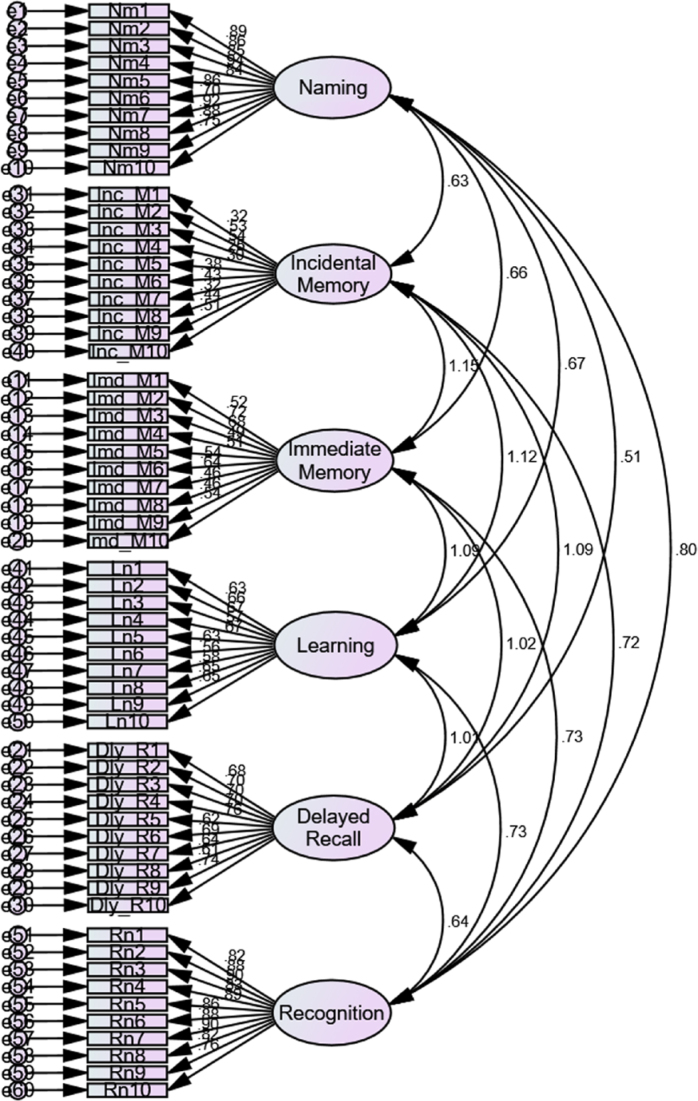
Structural model of the confirmatory factor analysis.

According to the scale validity test, most items had strong loading factors, with the exception of the Incidental Memory dimension. Item selection was based on standardized regression estimates, using a threshold of Loading Factor ≥0.50. As shown in Figure 1 and Table 4, nearly all dimensions contained valid items according to their loading factors; however, the Incidental Memory and Immediate Memory dimensions included several items that did not meet this criterion. Specifically, the Incidental Memory items Inc_M1, Inc_M4, Inc_M5, Inc_M6, Inc_M7, Inc_M8, and Inc_M9 had loading factors ranging from 0.254 to 0.439, indicating insufficient strength. Meanwhile, Immediate Memory items Imd_M4, Imd_M8, and Imd_M9 had loading factors between 0.464 and 0.489, which, while tolerable, were still below the ideal threshold.

**Table 4:** Confirmatory factor analysis.

Item	1	2	3	4	5	6
Nm1	0.894					
Nm2	0.859					
Nm3	0.850					
Nm4	0.936					
Nm5	0.841					
Nm6	0.858					
Nm7	0.702					
Nm8	0.920					
Nm9	0.875					
Nm10	0.747					
Imd_M1		0.519				
Imd_M2		0.716				
Imd_M3		0.676				
Imd_M4		0.491				
Imd_M5		0.508				
Imd_M6		0.540				
Imd_M7		0.642				
Imd_M8		0.465				
Imd_M9		0.464				
Imd_M10		0.537				
Dly_R1			0.678			
Dly_R2			0.705			
Dly_R3			0.704			
Dly_R4			0.700			
Dly_R5			0.757			
Dly_R6			0.619			
Dly_R7			0.690			
Dly_R8			0.636			
Dly_R9			0.605			
Dly_R10			0.742			
Inc_M1				0.324		
Inc_M2				0.526		
Inc_M3				0.542		
Inc_M4				0.254		
Inc_M5				0.304		
Inc_M6				0.383		
Inc_M7				0.426		
Inc_M8				0.320		
Inc_M9				0.439		
Inc_M10				0.513		
Ln1					0.628	
Ln2					0.663	
Ln3					0.672	
Ln4					0.568	
Ln5					0.666	
Ln6					0.630	
Ln7					0.559	
Ln8					0.577	
Ln9					0.652	
Ln10					0.647	
Rn1						0.818
Rn2						0.885
Rn3						0.901
Rn4						0.831
Rn5						0.895
Rn6						0.858
Rn7						0.882
Rn8						0.895
Rn9						0.816
Rn10						0.757

## DISCUSSION

In this study we analyzed the psychometric properties of BCSB-INA in older adults. The BCSB-INA emerged as an alternative to address the specific needs of Indonesia's diverse population. Traditional cognitive assessments often rely heavily on verbal skills and assume a certain educational background^6^, which may not be applicable to all demographic groups in Indonesia. The BCSB-INA demonstrates good psychometric properties, with high internal consistency and acceptable construct validity. The CFA results, while overall positive, suggest that certain dimensions —particularly "Incidental Memory" — could benefit from further refinement. This refinement may involve adjusting their phrasing to better capture the intended construct. We found high reliability in the psychometric evaluation of the BCSB-INA, with a Cronbach's alpha of 0.968, indicating that the instrument is consistent in measuring cognitive abilities throughout its various domains. Sufficient reliability is crucial for ensuring strong validity and should be assessed during the validation of an instrument. Other researchers evaluating brief cognitive tests have also reported good reliability, which was generally higher than 0.7^17^.

To contextualize the psychometric performance of the BCSB-INA, we drew comparisons to other established cognitive screening tools used in diverse populations. For example, the Japanese version of the Montreal Cognitive Assessment (MoCA-J) demonstrated strong internal consistency (Cronbach's alpha=0.74), high test-retest reliability (0.88), and excellent sensitivity and specificity in detecting mild cognitive impairment and Alzheimer's disease, making it a valuable screening tool for clinical and community settings^18^. Likewise, the Dementia Screening Battery-100 (DSB-100) was developed as a culturally-appropriate dementia screening tool for Tunisian older adults. It showed high interrater reliability when tested on demented individuals and healthy controls, validating its use in non-Western populations^19^. These comparisons emphasize the importance of developing culturally- and linguistically-appropriate cognitive tools, such as the BCSB-INA, to effectively meet the needs of underrepresented populations. We found that the mean scores throughout different cognitive dimensions showed variation, with the highest scores observed in the "Recognition" dimension (8.94±2.685) and the lowest scores in the "Incidental Memory" (4.21±2.294). This indicates that participants generally performed better on tasks related to recognition compared to tasks involving incidental memory. This finding is in line with previous studies and theoretical accounts for age-related differences in memory performances in older adults^20,21^.

The significant positive correlation between the BCSB-INA and the MoCA-INA further supports the convergent validity of the BCSB-INA. The correlation coefficients indicate that both tools effectively measure similar cognitive constructs, reinforcing the BCSB-INA's role as a viable alternative for cognitive assessment in the Indonesian context. The positive correlations observed among various dimensions of both instruments suggest that the BCSB-INA can reliably distinguish between different levels of cognitive impairment. Authors of a previous study also found that the delayed recall of BCSB had a significant correlation with the Rey Auditory Verbal Learning Test (RAVLT) in a study on healthy older adults^22^. Authors of a review on the use of BCSB in various clinical settings also showed good psychometric properties of BCSB both as a screening tool and as a diagnostic assessment^16^.

### Implications for practice and future research

The development of the BCSB-INA represents a critical step toward improving dementia diagnosis and care in a culturally-sensitive manner. As healthcare providers become more aware of the importance of early detection, the BCSB-INA can serve as a valuable tool for identifying individuals at risk for dementia and facilitating timely intervention.

Future research should focus on further evaluating the BCSB-INA's effectiveness in distinguishing between cognitively healthy individuals and those with cognitive impairments. Studies assessing the tool's predictive validity over time will provide deeper insights into its utility in clinical settings. Moreover, exploring the experiences of healthcare providers using the BCSB-INA in practice can inform ongoing improvements and adaptations to the tool.

All in all, the BCSB-INA offers a culturally- and linguistically-appropriate cognitive assessment tool for diagnosing dementia in Indonesia, addressing an urgent need in the field. With strong psychometric properties and cultural relevance, it enhances early detection and management of cognitive impairments, crucial for improving healthcare outcomes in an aging population. Ongoing refinement and research will be vital to maximizing its effectiveness in addressing dementia in Indonesia and beyond.
